# Guillain–Barre syndrome of acute motor axonal neuropathy (AMAN) type associated with herpes zoster: a case report

**DOI:** 10.1186/s12883-024-03607-1

**Published:** 2024-04-03

**Authors:** Leonard Wanninayake, Dilani Rajapaksha, Narmada Nair, Kamal Gunarathne, Udaya Ranawaka

**Affiliations:** 1 Department of Pharmacology, Faculty of Medicine, University of Colombo, Colombo, Sri Lanka; 2 Colombo North Teaching Hospital, Ragama, Sri Lanka; 3 National Hospital of Sri Lanka, Colombo, Sri Lanka; 4 Department of Medicine, Faculty of Medicine, University of Kelaniya, Ragama, Sri Lanka

**Keywords:** AMAN, Guillan Barre Syndrome (GBS), Herpes zoster

## Abstract

Guillain Barre syndrome (GBS) following Varicella zoster is a rare presentation and has only been reported in a few cases around the world. Of the reported cases, the type of GBS is not specified in the majority, and where specified is of the acute inflammatory demyelinating polyradiculoneuropathy (AIDP) type. We report a case of acute motor axonal neuropathy (AMAN) type GBS following herpes zoster in a 27-year-old male who presented with bilateral lower limb weakness and left sided lower motor neuron type facial nerve palsy a week after herpes zoster infection.

## Introduction

Guillain–Barre syndrome (GBS) is a relatively rare disorder with an incidence of 1–2 per 100,000 cases [1]. It is an acute peripheral neuropathy with several subtypes described based on clinical and electrophysiological features: acute inflammatory demyelinating polyradiculoneuropathy (AIDP), acute motor axonal neuropathy (AMAN), acute motor and sensory axonal neuropathy (AMSAN), acute sensory neuropathy, acute pandysautonomia and overlap syndrome [2]. The prevalence of different types of GBS varies with geographic location [2].

Several infectious triggers have been implicated in the pathogenesis of GBS. Of them, the most common is *Campylobacter jejuni* infection which is commonly associated with AMAN type GBS [3]. Several other infectious agents such as Epstein Barr virus, *Mycoplasma pneumoniae*, Cytomegalovirus and HIV have been implicated as common aetiologies. GBS can rarely manifest following some vaccines such as measles, rabies, influenza, measles and MMR [2]. More recently, GBS has been reported following SARS-CoV-2 infection and COVID-19 vaccination [4, 5]

Acute varicella infection (chicken pox) is a rare trigger for GBS [3], and GBS following varicella infection is usually of the AIDP type [6–9]. A recent review identified 88% of the GBS cases following varicella infections to be of the AIDP type, and 13% axonal type [6, 7, 10–28]. Herpes zoster is a clinical syndrome due to reactivation of dormant varicella virus in sensory ganglia several years after initial varicella infection [10]. GBS following herpes zoster is a rare association described only in a few case reports [6–8, 10, 29–34]; where reported, it has always been of the AIDP variety [6, 29–36]. We were unable to identify any reports of zoster-associated AMAN type GBS on a literature survey of several electronic databases although a few cases of AMAN type GBS after chicken pox have been reported. We report a case of AMAN type GBS associated with herpes zoster.

## Case report

**Fig. 1:**
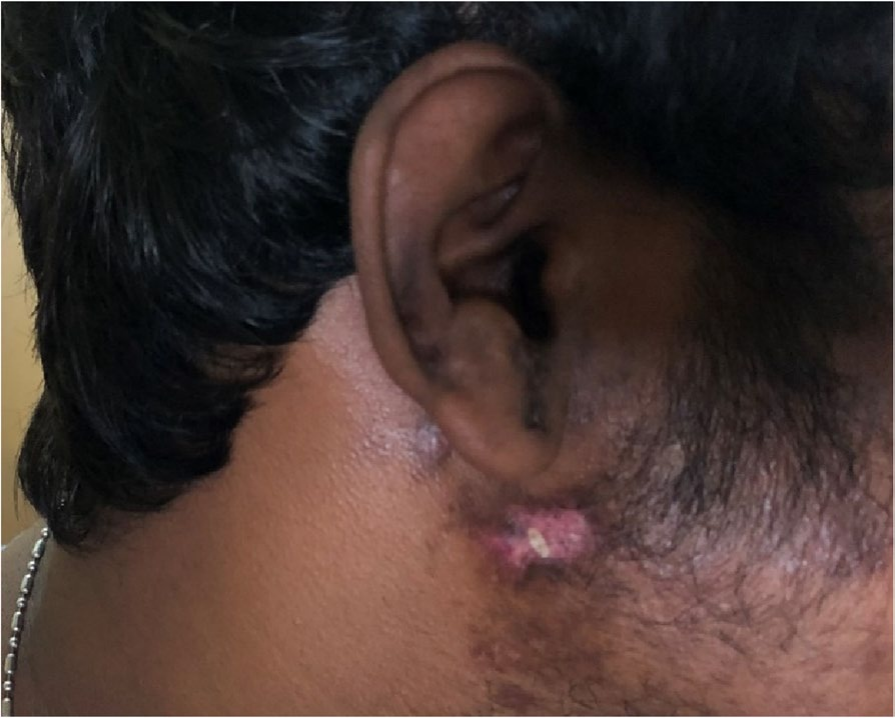
Herpes zoster rash over the right face

A 27-year-old previously healthy man initially presented with a painful vesicular skin rash over the maxillary and mandibular distributions of the right trigeminal nerve (as shown in Fig. 1), which was clinically diagnosed as herpes zoster and treated at the local hospital outpatient clinic with oral acyclovir. Two days following the onset of the rash, he developed progressive bilateral lower limb weakness with predominant difficulty in standing up from a seated position. Six days later, he developed progressive bilateral upper limb weakness, difficulty with left eyelid closure and deviation of the mouth to the right side. There was no associated incoordination, or difficulty in speech or swallowing. He was admitted to a Sri Lankan tertiary care hospital on the 7th day of the illness. He was an unmarried driver, who was independent in his daily activities prior to the onset of the current illness. There was no history of smoking, alcohol use or substance abuse.

On admission, there was no fever, pallor, jaundice or lymphadenopathy. No evidence of muscle wasting or fasciculations was noted. Lower limb neurological examination revealed bilateral hypotonia and predominant proximal weakness, with MRC grade 3 power in hip flexors and extensors, and MRC grade 4- power in the flexors and extensors at the knee and ankle. Bilateral knee and ankle tendon reflexes were diminished (grade 1/4) with flexor plantar responses. Upper limb neurological examination showed normal tone bilaterally with MRC grade 4 + power in all muscle groups at shoulder, elbow and wrist, and normal biceps and triceps tendon reflexes. Cranial nerve examination revealed a left sided lower motor neuron type facial nerve palsy. No other cranial nerve abnormalities or cerebellar signs were noted. Sensory examination of the face, lower limbs and upper limbs was normal for pain, temperature, touch and proprioception.

Routine investigations including full blood count, electrolytes, creatinine, ESR, CRP, liver function tests, TSH and fasting blood sugar were normal. Retroviral screening was negative. MRI scan of the lumbosacral spine was normal. Nerve conduction studies (NCS) and EMG revealed AMAN type GBS (Table 1). CSF analysis showed elevated protein of 78 mg/dL, with 12 lymphocytes/mm3 and no neutrophils. CSF glucose was 76.5 mg/dl with a simultaneous blood glucose of 100 mg/dl. CSF testing for ADA, TB Gene expert and TB culture were negative. Serology for varicella zoster IgG antibodies was positive after 2 weeks from presentation, whereas varicella IgM antibodies were equivocal.

**Table 1:** Nerve conduction and EMG findings of Case

**Motor nerve conduction**						
Nerve and site	Latency	Amplitude	Segment	Latency difference	Distance	Conduction velocity
Right Peroneal						
Ankle	4.4 ms (< 6.5 ms)	0.8 mV (> 1.3 mV)	Extensor digitorum brevis (EDB) – ankle			
Fibular head	12.7 ms	0.8 mV	Ankle—fibular head	8.3 ms	290 mm	34.9 m/s (> 38 m/s)
Right Tibial						
Ankle	5.9 ms (< 6.1 ms)	2.2 mV (> 4.4 mV)				
Left peroneal nerve						
Ankle	4.2 ms (< 6.5 ms)	1.0 mV (> 1.3 mV)	EDB—ankle			
Fibular head	12.5 ms	0.4 mV	EDB—fibular head	8.3 ms	300 mm	36.1 m/s (> 38 m/s)
Right Ulnar						
Wrist	2.6 ms (< 3.7 ms)	2.2 mV (> 7.9 mV)				
Below elbow	9.1 ms	1.6 mV	Wrist—below elbow	6.5 ms	180 mm	47.0 m/s (> 52 m/s)
F wave studies						
Nerve		M-Latency		F-Latency		
Right tibial		9.5 m/s		34.6 m/s (50.8 m/s), Repeaters present, Reduced persistence		
**Sensory nerve conduction**						
Nerve and site		Onset latency	Peak latency	Amplitude	Segment	Latency difference
Right sural nerve at lower leg		2.8 ms (3.6 ms)	3.3 ms (4.5 ms)	6.9 uV (4 uV)	Ankle lower leg	2.8 ms
Right ulnar at 5th Digit		2.0 ms (3.1 ms)	2.3 ms (4 ms)	11.8 uV (6 uV)	Wrist at 5th Digit	2.0 ms
**EMG**		Right rectus femoris: Poor activation and large motor units. Right tibialis anterior: Reduced recruitment and high firing. Features are consistent with denervation in these muscles. No evidence of myopathy or myositis				

Given the close temporal relationship to herpes zoster infection, a diagnosis of GBS secondary to herpes zoster was made. He was treated with intravenous immunoglobulin [IVIg] 2 g/kg divided over 5 days duration and regular physiotherapy. Limb and facial weakness gradually improved after one week of hospital stay and he was discharged with plans for outpatient rehabilitation. At the time of discharge, he had MRC grade 4 + power in all four limbs. On three months review, he had normal limb power in all muscle groups (MRC 5) and normal facial muscle strength.

## Discussion

Herpes zoster and varicella zoster manifests with several sensory and motor manifestations. Transverse myelitis, varicella associated segmental motor weakness, Ramsey hunt syndrome, GBS are some of many motor manifestations of herpes zoster [37]. We report a case of GBS following herpes zoster, which adds to the limited number of cases in published literature. The neurophysiological subtype of GBS has not been reported in most published cases of zoster-related GBS. Where specified, it had been of the acute inflammatory demyelinating polyradiculoneuropathy (AIDP) type [6–9]. Our patient had GBS of the acute motor axonal neuropathy (AMAN) type. To the best of our knowledge, this is the first case of AMAN type GBS confirmed with electrodiagnosis, associated with herpes zoster.

Herpes zoster is known to be associated with several motor and sensory neurological manifestations such as post herpetic neuralgia, meningoencephalitis, myelitis, poly-cranial neuritis, motor dysfunction and GBS. The pathophysiology of zoster-related GBS is not very well understood. The postulated hypotheses are molecular mimicry or the deranged immune system in the host, particularly of the lymphocyte subtypes. Although molecular mimicry with *C. jejuni* has been demonstrated, molecular mimicry with varicella zoster and peripheral nerves has not been confirmed [38, 39].

In the majority of reported cases, the diagnosis had been based on clinical grounds with the chronological relationship between the rash and neurological symptoms [33, 34]. There had been a few case reports with positive varicella serology in CSF and serum but with negative varicella PCR [22, 29]. Some reports have considered reduced CD 8 lymphocyte response as an evidence of varicella zoster infection [10]. Our patient had positive IgG varicella serology but equivocal IgM results. This is likely to be the result of delayed testing for serology as the hospital laboratory was being primarily utilized for COVID 19 testing during this time.

The delay to the onset of GBS symptoms following the zoster rash has varied from 3 to 42 days in previous cases [6, 29–36]. Our patient developed neurological manifestations 2 days after the rash. A majority of the reported cases of zoster related GBS (71%) had cranial nerve abnormalities, with the facial nerve involvement being the most common [6, 22, 33]. Our patient had lower motor neurone type facial nerve palsy, but no other cranial nerve abnormalities were noted. Some of the patients with zoster-related GBS had developed complications such as respiratory failure, and some case fatalities have been reported [6]. Mechanical ventilation was required in a few cases [6, 36]. However, our patient did not require intensive care or respiratory support and made a good recovery with treatment.

## Conclusion

We report a case of AMAN type GBS following herpes zoster. We believe our report adds to the growing literature on the diverse neurological complications following herpes zoster. Although rare, herpes zoster should be considered as an important underlying aetiological cause of GBS.

## Data Availability

All data and the materials of the patient are available with the corresponding author.
